# A Potential Benefit of “Balanced Diet” for Rheumatoid Arthritis

**DOI:** 10.3389/fmed.2018.00141

**Published:** 2018-05-15

**Authors:** Kayo Masuko

**Affiliations:** ^1^Health Evaluation and Promotion Center, Sanno Medical Center, Tokyo, Japan; ^2^Clinical Research Center for Medicine, International University of Health and Welfare, Tokyo, Japan

**Keywords:** diet, rheumatoid arthritis, gut microbiota, nutrition, carbohydrate, omega-3 polyunsaturated fatty acids

## Abstract

Although it is largely unknown how diet might modulate rheumatoid arthritis (RA), dietary interventions, including so-called “low-carbohydrate” diets, may be considered for RA patients because of the high incidence of cardiovascular comorbidity. However, it has been shown that restriction or skewed intake of particular nutrient may alter the components of the intestinal flora. Changes to the gut microbiota or dysbiosis may be relevant to the pathogenesis of RA because the gut microbiota is reported to regulate the T cell phenotype and T cell-mediated immunity. RA patients should be advised that a balanced diet that includes appropriate amounts of carbohydrate, especially dietary fiber, is important for maintaining the symbiosis of intestinal flora, which could be beneficial for preventing autoimmunity. The review attempts to focus current findings regarding the suggested relationship between diet-derived carbohydrate, gut microbiota, and the pathogenesis of RA.

## Introduction

There has been a long-standing debate, which has continued even since the development of remission-inducible disease-modifying anti-rheumatic drugs (DMARDs), over whether diet plays a pathogenic or a disease-modulating role in autoimmune diseases including rheumatoid arthritis (RA) (1, 2). Various dietary patterns, such as Mediterranean-style or vegetarian diets, as well as nutritional factors including polyphenols and omega-3 polyunsaturated fatty acids are speculated to be relevant to the occurrence and/or outcome of the disease. However, none of these diet or nutrients has been established so far as bringing substantial benefit with a clinically proven disease-modifying effect (3, 4).

On the other hand, studies have shown that the risk of metabolic syndrome is higher in RA patients than in healthy subjects (5). Further, RA patients may be in condition so-called “rheumatoid cachexia,” that is, they would have an accumulation of adipose tissue besides decrease of muscle (i.e., sarcopenia) due to catabolic change and decreased physical activity (5, 6). Such metabolic imbalance in RA is caused by chronic inflammation with elevated levels of wasting cytokines including tumor necrosis factor (TNF)-α, therefore potent anti-rheumatic therapy using DMARDs may improve the imbalance at least to some extent (6, 7). Nevertheless, even if the inflammation would be controlled by the medication, appropriate nutritional intervention should still be considered for these patients, who may have a skewed nutritional intake and insufficient quality of diet (8). In fact, we have observed that some RA patients have difficulty in cooking and/or eating because of their arthritic symptoms, and that the limitations in their joint movement may affect their dietary intake (9).

Omega-3 PUFAs (such as eicosapentaenoic acid and docosahexaenoic acid) have been reported to exert an anti-inflammatory effect through intra-nuclear signals that suppress inflammatory signals via peroxidase proliferator activator receptors (10). As for RA, there are studies that showed the efficacy of omega-3 PUFAs or fish oil (as a source of omega-3 PUFA) for reducing RA-related disease activity markers, while improving the blood lipid profile [reviewed in (11)]. Nevertheless, statistical significance of these experiments in which PUFAs were administered to RA patients are not essentially robust because of its sample size and/or clinical setting, as there are also reports with negative results (12, 13); instead, long-term administration of relatively high dose of PUFA in RA patients might cause adverse events such as critical hemorrhage (14). Solid clinical evidence should be obtained from large-scale, placebo-controlled double-blinded clinical trials, although this may be difficult to achieve, because the clinical background and dietary habit differ among patients.

Of note, from a nutritional view, excess consumption of any oil or fat will also increase total energy intake (i.e., the total number of calories consumed each day). As diet does not involve only the serial ingestion of a single nutrient, the relative proportions of protein, fat, and carbohydrate intake may also alter that individual's overall health outcome, including the immunological responses (15).

## Gut microbiota modulate immunity

From the oral cavity to the rectum, the gastrointestinal tract can be exposed to extrinsic pathogens. The largest immune system in the body, the gut-associated lymphatic tissue, has evolved in the gut mucosa under the coexistence of microbiota on the gut surface to protect the body from pathogenic attack (16). This tissue includes Peyer's patches in the small intestine and mesenteric lymph nodes, which trigger immune responses against antigens that enter the intestine.

Around 100 trillion bacteria are thought to be living in the human gastrointestinal tract, and the composition of the bacteria (“gut microbiome”) varies under influence of many factors including diet, medication, smoking or stress (17, 18).

The commensal gut bacteria have an important impact to individual's immune responses, both of innate and adoptive systems. For the initial defense against gut epithelium-invading pathogen, pattern-recognition receptors including toll-like receptors (TLRs) recognize microorganism-associated molecular patterns (MAMP) like flagellin and lipopolysaccharide (LPS). Intracellular signals via TLRs, e.g., TLR4 expressed in intestinal epithelial cells, would then lead to expression of pro- or anti-inflammatory cytokines. Indigenous microbiota of gram-positive rods, Clostridia and Bifidobacteria for example, regulate immunity by distinct responses; *Clostridium difficile* was reported to recruit neutrophils to the infected sites to promote inflammation, while *Bifidobacterium infantis* suppresses inflammation by inducing anti-inflammatory cytokines like interleukin (IL)-10 from dendritic cells (18).

Gut bacteria can also regulate T cell differentiation, thereby modulating adoptive immunity. For instance, Clostridia were found to induce expression of transforming growth factor (TGF)-β, which would lead to regulatory T cell (Treg) differentiation to maintain homeostasis of gut environment (19–21). The anti-inflammatory effect of Clostridia was supported by an experiment in which oral administration of the bacteria attenuated experimental colitis with a shift of T cells to anti-inflammatory subset (20).

Nutrients, in particular dietary fiber, is an important regulator of the gut microbiome (18, 21, 22). During fermentation of dietary fiber, gut bacteria release short-chain fatty acids (SCFAs) (including acetate, butyrate, and propionate), which synergistically act to facilitate gut homeostasis with the dietary fiber (21). For example, besides the promotion of Treg cell responses, SCFAs are known to reduce expression of inflammatory chemokines or cytokines such as TNF-α, IL-6, and interferon-gamma (21).

## Gut microbiota and RA

The potential implication of gut or oral microbiome in rheumatic diseases had been a debate for a long time, and recent development of experimental technology, for example, sequencing, analysis of molecular markers, and the “omics” methodologies has greatly advanced the knowledge [reviewed in (23)].

Accumulating studies demonstrated that gut microbiome of RA patients have distinct gut microbiota compared to control diseases or healthy subjects. Although the unique microbiome might be a secondary effect to the disease and may not necessarily reflect causal relationship (24), studies obtained from early stage or DMARDs-naïve RA patients seem to reflect more disease-related microbiome. In this regard, an imbalance of Prevotella species in intestinal microbiome have been found in studies with early-stage RA patients (23, 25, 26). Although its pathological role has not been understood, further investigation will clarify how the biased microbiome would contribute to the pathophysiology of RA. Interestingly, abundant presence of Prevotella has been suggested to correlate with a presence of “shared-epitope” genotype in RA, implying that potential contribution of a particular human leukocyte antigen (HLA) genotype in the formation of unique microbiome and also in the susceptibility of autoimmune disease (23).

As RA is an autoimmune disease, it can be speculated that there would be molecular similarity between bacterial component(s) and potential autoantigen(s) (i.e., “molecular mimicry”), and that T cell recognition of the bacterial component might trigger the pathogenic autoimmunity [reviewed in (27)].

Using a metagenomic survey, Zhang et al. (28) reported that gut samples from RA patients were enriched in Gram-positive bacteria and depleted of Gram-negative bacteria; and demonstrated an existence of molecular mimicry between RA-associated antigens (e.g., collagen XI, HLA-DRB1) and microbial genes (e.g., from Clostridia) identified in gut and oral samples from RA patients. Further, Pianta et al. (29) demonstrated a sequence homology between two newly identified RA-specific autoantigens (N-acetylglucosamine-6-sulfatase and filamin A) and gut bacteria (*Prevotella* species), demonstrating cross-reactivity to the microbial peptides by T cells from RA patients.

## “Diet and RA”: the balance determines

A recent paper by Hu et al. reported an interesting correlation between diet and RA (30). The authors evaluated the association between long-term dietary quality, measured according to the 2010 Alternative Healthy Eating Index (AHEI-2010), and the incidence of RA. The AHEI-2010 scores dietary quality based on an individual's intake of “healthy” foods (such as fruit, vegetables, whole grains, nuts, omega-3 fatty acids, PUFAs, and moderate alcohol consumption) and “unhealthy” items (including sugar-sweetened beverages, red and processed meat, trans fats, and sodium). Higher scores were positively associated with a “desirable lifestyle,” with reduced risk for chronic diseases such as cardiovascular diseases and type 2 diabetes. The study found that women with higher AHEI-2010 scores had a lower risk of developing RA. More specifically, women with scores in the top quartile had a 33% lower risk of RA compared to those with scores in the lowest quartile. Based on the finding, the authors concluded “an overall healthy diet quality may be more beneficial for RA risk reduction than individual foods and nutrients, particularly for early-onset seropositive RA” (30). Although the mechanisms underlying this protection have not been determined, it may result from a higher intake of antioxidants and dietary fiber (30). Indeed, intake of dietary fiber has been reported to ameliorate inflammation (31), whereas its deficiency enhances inflammation through degradation of mucosal barrier of intestine (32).

It has been reported that enterotypes (clusters of a relatively stable gut microbiome) may be determined or influenced by long-term dietary patterns (33, 34). dominance of *Prevotella* and *Bacteroides* species could be associated with dietary intake: a higher intake of animal protein and saturated fat would be associated with a dominance of *Bacteroides*, whereas a higher carbohydrate intake would favor *Prevotella* dominance (33). As previously mentioned, *Prevotella* is one of the candidate pathogenic bacteria for human RA. If so, a higher carbohydrate intake may lead to alteration of gut microbiome such that *Prevotella* species dominate, thereby increasing susceptibility to RA. In turn, the reported efficacy of omega-3 fatty acids against RA may partly depend on the suppression of *Prevotella*-induced inflammation (35).

**Figure 1:**
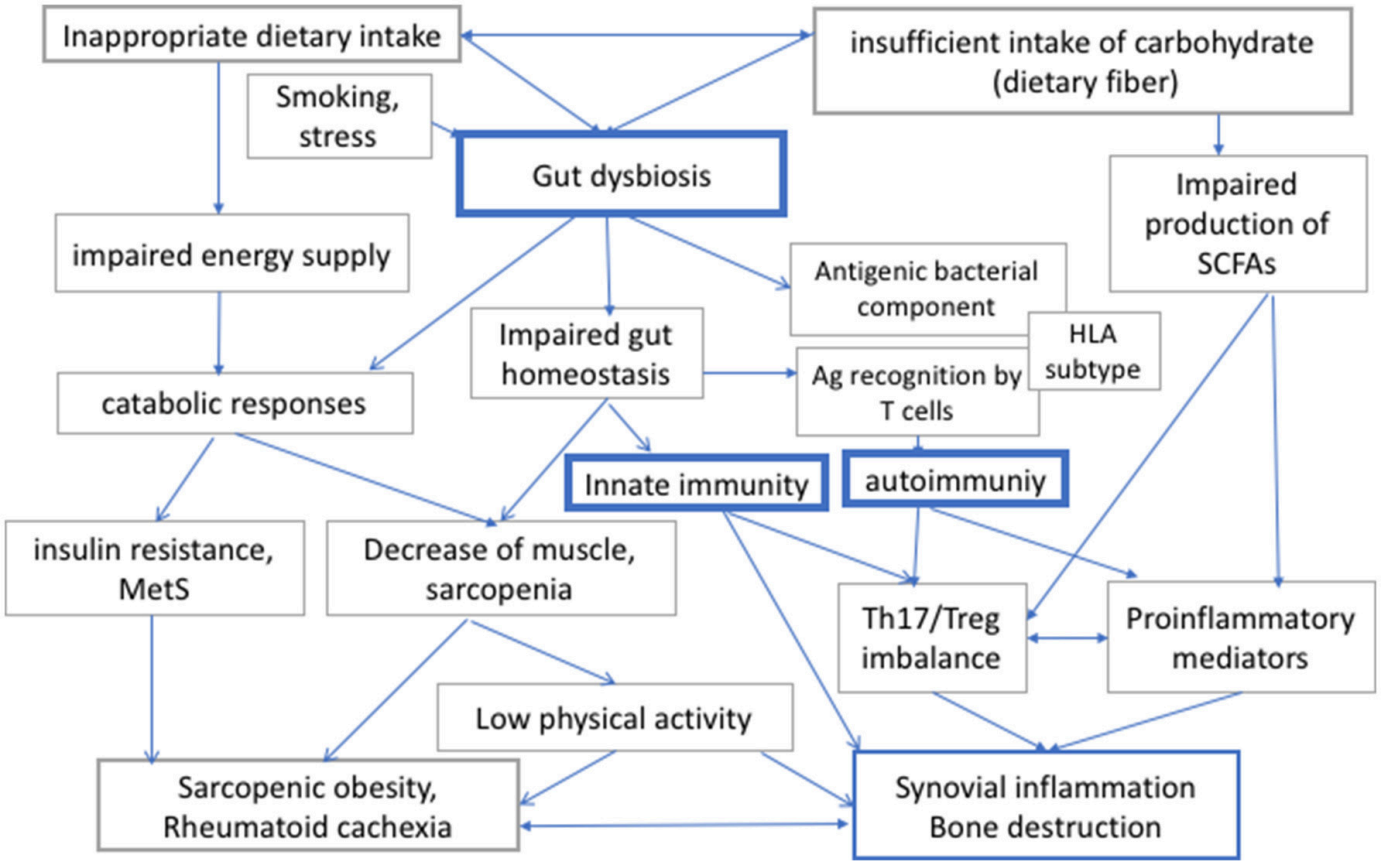
The interaction between diet, gut dysbiosis, and immune responses in rheumatoid arthritis (RA). Human gut microbiome regulates local and systemic immune responses, and its bacterial composition can be modulated by diet and other environmental factors. Diet is also important both for energy supply for physical activities. Therefore, insufficient or inappropriate dietary intake, or intake of carbohydrates, which is a source of energy and dietary fiber, would affect both metabolic integrity and immune responses via modulation of gut microbiome in RA. RA, rheumatoid arthritis; Th17, helper T 17 cells; Treg, regulatory T cells; MetS, metabolic syndrome; SCFAs, short chain fatty acids; HLA, human leucocyte antigen.

Patients with RA may exhibit increased resting energy expenditure because of chronic inflammation, and the inflammation-induced metabolic imbalance would lead to rheumatoid cachexia (6). On the other hand, insufficient relative intake in carbohydrate intake leads to an excessive intake of fat and protein, which may result in the further deterioration of cardiovascular risks as well as in renal complications. Taken together, RA patients should be recommended not to consume excessive amounts of energy (i.e., calories) and carbohydrate, but they should nevertheless consume sufficient diet, including an appropriate amount of dietary fiber, to secure intake of appropriate energy and to maintain gut homeostasis (Figure 1).

## Concluding remarks

Along with smoking or stress, diet is one of the modifiable environmental factors for the onset and/or disease outcome of RA (36). Through improving metabolic imbalance, and via modulation of the gut microbiota, diet may gradually adjust the physiological condition as well as immunological response in RA patients.

A balanced intake of a variety of foodstuff including dietary fiber is important for maintaining diverse intestinal flora, and for reducing metabolic and inflammatory risks. Long-term restriction of a single nutrient may not be advisable and should only be done under careful observation by nutritional experts.

## Author contributions

KM conceived of the article, wrote, revised and approved the whole manuscript.

### Conflict of interest statement

The author declares that the research was conducted in the absence of any commercial or financial relationships that could be construed as a potential conflict of interest.
